# A spider derived peptide, PnPP-19, induces central antinociception mediated by opioid and cannabinoid systems

**DOI:** 10.1186/s40409-016-0091-6

**Published:** 2016-12-21

**Authors:** Daniela da Fonseca Pacheco, Ana Cristina Nogueira Freitas, Adriano Monteiro C. Pimenta, Igor Dimitri Gama Duarte, Maria Elena de Lima

**Affiliations:** 1 Departmento de Farmacologia, Instituto de Ciências Biológicas, Universidade Federal de Minas Gerais (UFMG), Belo Horizonte, MG Brazil; 2 Departamento de Bioquímica e Imunologia, Instituto de Ciências Biológicas, Universidade Federal de Minas Gerais (UFMG), Av. Antônio Carlos, 6627, Belo Horizonte, MG CEP 31.270.901 Brazil

**Keywords:** Peptide PnPP-19, Central antinociception, *Phoneutria nigriventer*, μ-opioid receptor, δ-opioid receptor, CB1 receptor, CB2 receptor

## Abstract

**Background:**

Some peptides purified from the venom of the spider *Phoneutria nigriventer* have been identified as potential sources of drugs for pain treatment. In this study, we characterized the antinociceptive effect of the peptide PnPP-19 on the central nervous system and investigated the possible involvement of opioid and cannabinoid systems in its action mechanism.

**Methods:**

Nociceptive threshold to thermal stimulation was measured according to the tail-flick test in Swiss mice. All drugs were administered by the intracerebroventricular route.

**Results:**

PnPP-19 induced central antinociception in mice in the doses of 0.5 and 1 μg. The non-selective opioid receptor antagonist naloxone (2.5 and 5 μg), μ-opioid receptor antagonist clocinnamox (2 and 4 μg), δ-opioid receptor antagonist naltrindole (6 and 12 μg) and CB_1_ receptor antagonist AM251 (2 and 4 μg) partially inhibited the antinociceptive effect of PnPP-19 (1 μg). Additionally, the anandamide amidase inhibitor MAFP (0.2 μg), the anandamide uptake inhibitor VDM11 (4 μg) and the aminopeptidase inhibitor bestatin (20 μg) significantly enhanced the antinociception induced by a low dose of PnPP-19 (0.5 μg). In contrast, the κ-opioid receptor antagonist nor-binaltorphimine (10 μg and 20 μg) and the CB_2_ receptor antagonist AM630 (2 and 4 μg) do not appear to be involved in this effect.

**Conclusions:**

PnPP-19-induced central antinociception involves the activation of CB_1_ cannabinoid, μ- and δ-opioid receptors. Mobilization of endogenous opioids and cannabinoids might be required for the activation of those receptors, since inhibitors of endogenous substances potentiate the effect of PnPP-19. Our results contribute to elucidating the action of the peptide PnPP-19 in the antinociceptive pathway.

## Background

PnPP-19 is a synthetic peptide that contains 19 amino acid residues [1]. It represents a part of the primary structure of the native toxin PnTx2-6, also known as δ-ctenitoxin-Pn2a [2], which was isolated from the venom of the spider *Phoneutria nigriventer* [3]. Some peptides purified from the venom of this spider have been identified as potential sources of drugs for pain treatment. For example, PnTx3-3, renamed ω-ctenitoxin-Pn2a [2], showed an antinociceptive effect in different models of neuropathic pain [4]. Additionally, Phα1β neurotoxin, another toxin isolated from that same venom, induced antinociception in models of neuropathic and inflammatory pain [5].

Cannabinoids and opioids are two separate groups of psychoactive drugs that exhibit several similar pharmacological effects, including analgesia, sedation, hypothermia and inhibition of motor activity [6–8]. In recent years, our group has demonstrated the involvement of endogenous opioids and cannabinoids in the antinociceptive action of several substances [9, 10]. Receptors for both drugs are coupled to similar intracellular signaling mechanisms and the interaction between cannabinoid and opioid systems in the nociceptive pathway has been the focus of much attention [9, 11–15].

Interestingly, it has been shown that endogenous opioids are involved in antinociception induced by a scorpion toxin [16]. Therefore, it is hypothesized that pain relief induced by alpha- or beta- scorpion toxins may implicate the activation of an endogenous opioid system. The analgesic effect of those toxins might be partially due to the activation of diffuse noxious inhibitory controls of supra-spinal origin, which are linked to a counter-irritation phenomenon and release of endogenous opioids [16]. Thus, opioid peptides may be involved in the action mechanism of other toxins, particularly toxins from other arthropods, such as the spider *Phoneutria nigriventer*.

Recently we have shown that PnPP-19 induces antinociception in the peripheral nervous system [17]. We suggested that this effect is attributable to an inhibition of the neutral endopeptidase (neprilysin), which may lead to an increase of enkephalin levels and may cause activation of both μ- and δ-opioid receptors. In addition, we showed evidence that the receptor CB1 is implicated in the antinociceptive effect induced by PnPP-19.

Given the lack of information concerning the antinociceptive effect of PnPP-19 on the central nervous system (CNS), the aim of the present study was to determine the possible effect of this peptide on the CNS and investigate whether there is an involvement of the cannabinoid and opioid systems.

## Methods

### Animals

The experiments were performed on 25–30 g male Swiss mice (*n* = 4 per group) provided by the CEBIO (“Centro de Bioterismo”, the Animal Center) of the Universidade Federal de Minas Gerais (UFMG). The mice were housed in a temperature-controlled room (23 ± 1 °C) on an automatic 12-h light/dark cycle (06:00–18:00 h of light phase). All tests were carried out during the light phase (08:00–15:00 h). Food and water were freely available until the onset of the experiments. The algesimetric protocol was approved by the Committee for Ethics in Animal Experimentation (CETEA) of UFMG, with the protocol number 131/2014.

### Algesimetric method

The tail-flick test used in the present study was conducted in accordance with the procedure described by D^’^Amour and Smith [18] with a slight modification. The test consists of restraining the mouse by one of the experimenter’s hands and positioning the distal portion of the mouse’s tail (about 2 cm from the tip of the tail) under a helical nickel-chrome resistance. When the device is turned on, an electric current starts to flow through the resistance, which may lead to a rise of its temperature. In addition, the moment that the equipment is turned on, a timer is activated. The time required for the animal to perceive the nociceptive stimulus and execute the tail withdrawal reflex is measured and expressed in seconds. The intensity of the heat reached by the resistance was adjusted, so the baseline latencies required to observe the withdrawal reflex of the mouse’s tail were between 3 and 4 s (the thermal stimulus applied increased from 0.297 calories/s). To avoid tissue damage, the cutoff time was established at 9 s [19]. The baseline latency was obtained for each animal before drug administration (zero time) by calculating an average of three consecutive trials. To reduce stress, mice were habituated to the apparatus one day prior to conducting the experiments.

### Intracerebroventricular injection (i.c.v.)

Animals were constrained by an acrylic tube-shaped device (Insight, Brazil). To facilitate the i.c.v. injection, the animals were placed inside this tube, which immobilizes their body, except for their head. With one hand, the experimenter restrained the animal’s head and then injected the drugs into its right lateral ventricle, by the intracerebroventricular route, using a Hamilton syringe of 5 μL. The site of injection was 1 mm from either side of the midline of a line drawn through the anterior base of the ears (modified from Haley and McCormick, [20]). The syringe was inserted perpendicularly through the skull into the brain at the depth of 2 mm, and 2 μL of solution was injected. To determine whether drugs were injected correctly into the brain ventricular system, they were diluted in a solution containing Evans blue 0.5%. Once the experiment was finished, the animals were euthanized with an overdose of anesthesia and their brains were sectioned for confirmation of the side of injection.

### Experimental protocol

All drugs were i.c.v. administered into the right lateral ventricle. Naloxone, clocinnamox, naltrindole, nor-binaltorphimine, AM251, AM630, MAFP, VDM11 and bestatin were administered 1 min prior to administration of PnPP-19. The protocol to determine the best moment for the injection of each substance was assessed in pilot experiments and literature data [10, 15].

The nociceptive threshold was always represented by the time, in seconds, required for the animal to exhibit the tail withdrawal reflex. The measurements were performed before any drug administration and after 5, 10, 15 and 30 min after drug injection.

### Statistical analysis

Data were analyzed statistically by Repeated Measures ANOVA with post-hoc Bonferroni’s test for multiple comparisons. Probabilities less than 5% (p < 0.05) were considered to be statistically significant.

### Chemicals

The following drugs and chemicals were used: PnPP-19 (synthesized by China Peptides, China), the opioid receptor antagonist naloxone (Sigma, USA), the μ-opioid receptor antagonist clocinnamox (Tocris, USA), the δ-opioid receptor antagonist naltrindole (Tocris, USA), the κ-opioid receptor antagonist nor-binaltorphimine (Sigma, USA), the aminopeptidase inhibitor bestatin (Sigma, USA), AM251 [N-(piperidin-1-yl)-5-(4-iodophenyl)-1-(2,4-dichlorophenyl)-4-methyl-1H-pyrazole-3-carboxamide; Tocris, USA], AM630 {6-iodo-2-methyl-1-[2-(4-morpholinyl)ethyl]-1H-indol-3-yl(4-ethoxyphenyl) methanone; Tocris, USA}, MAFP [(5*Z*,8*Z*,11*Z*,14*Z*)-5,8,11,14-eicosatetraenyl-methyl ester phosphonofluoridic acid, Tocris, USA] and VDM11 [(5*Z*,8*Z*,11*Z*,14*Z*)-*N*-(4-Hydroxy-2-methylphenyl)-5,8,11,14-eicosatetraenamide, Tocris, USA].

The drugs were dissolved as follows: PnPP-19 (saline), naloxone (saline), clocinnamox (saline), naltrindole (saline), nor-binaltorphimine (saline), bestatin (saline), AM251 and AM630 (12% DMSO in saline), MAFP (10% DMSO in saline), VDM11 (10% in saline) and injected at a volume of 2 μL into the lateral ventricle. The saline used for dilution of all drugs contained 0.5% Evans Blue.

## Results

### Antinociceptive effect of PnPP-19

**Fig. 1:**
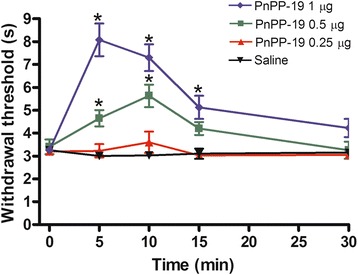
Central antinociception induced by intracerebroventricular administration of PnPP-19 in mice. PnPP-19 (0.25, 0.5 and 1 μg) was administered 5 min prior measurement in the tail-flick test. Each line represents the mean ± SEM for four mice per group. *Significant difference compared to the Saline-injected group (ANOVA + Bonferroni test, *p* < 0.05). Saline (0.5% of Evans Blue)

Since the peptide PnPP-19 is known to induce peripheral antinociception, we decided to investigate whether it could also interact with the central nervous system and induce antinociception mediated by activation of central signaling related to the nociceptive pathway [17]. Firstly, PnPP-19 was injected intracerebroventricularly. Then it was observed that the doses of 0.5 and 1 μg/per animal induced a significant delay of the tail withdraw reflex of the mice. This result may indicate that at those doses, PnPP-19 leads to an antinociceptive response in a dose-dependent manner (Fig. 1). The dose of 0.25 μg/per animal was ineffective whereas the control group of mice injected only with vehicle (saline) remained unaltered. The dose of 1 μg was chosen for the following experiments in the present study since it had almost reached the cutoff time of the test (9 s).

### Antagonism of PnPP-19-induced antinociception by naloxone, clocinnamox, naltrindole and AM251

**Fig. 2:**
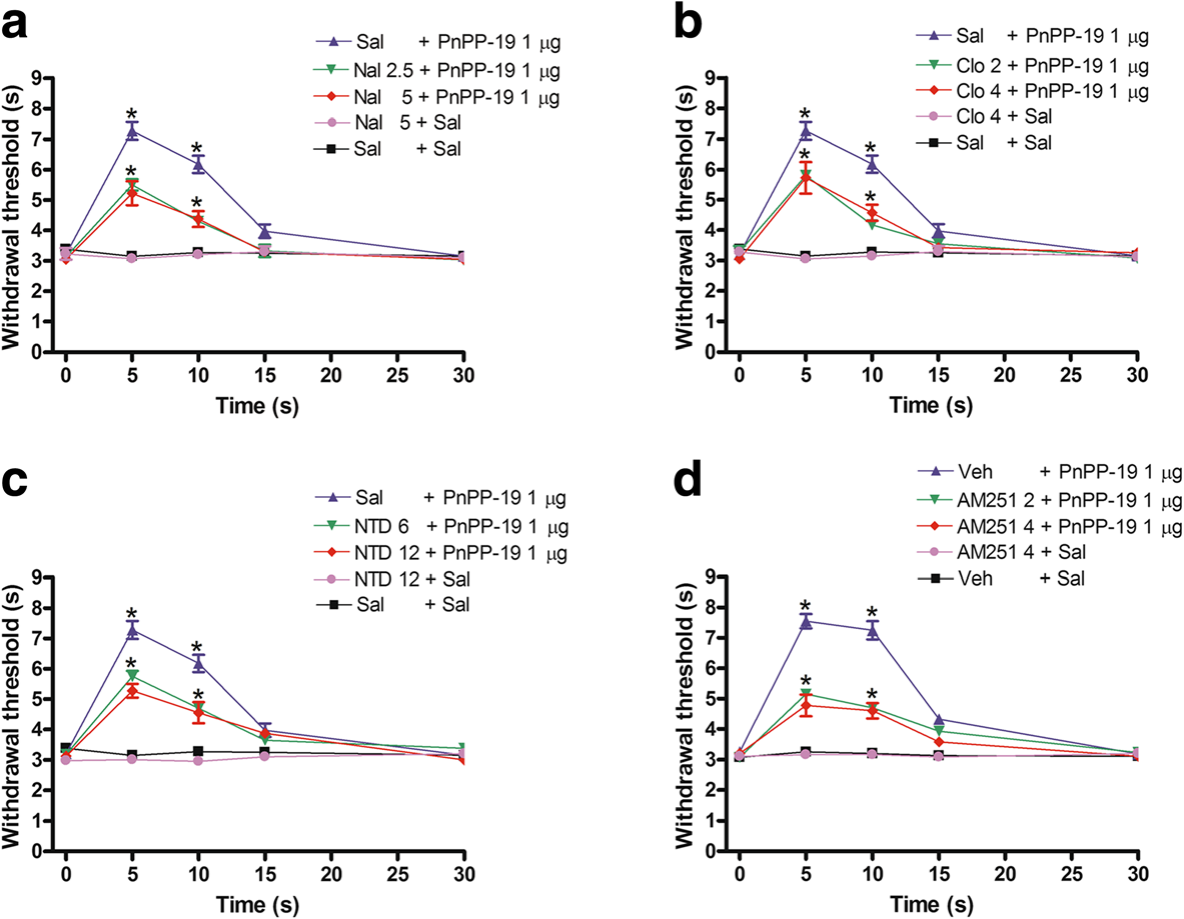
Partial antagonism induced by intracerebroventricular administration of **a** naloxone, **b** clocinnamox, **c** naltrindole or **d** AM251 in the central antinociception induced by PnPP-19. Naloxone (Nal; 2.5 and 5 μg), clocinnamox (Clo; 2 and 4 μg), naltrindole (NTD; 6 and 12 μg) or AM251 (2 and 4 μg) was administered 1 min prior to PnPP-19 injection (1 μg). These antagonists did not significantly modify the nociceptive threshold in the control group. Each line represents the mean ± SEM for four mice per group. *Significant difference compared to the control group (ANOVA + Bonferroni’s test). Sal: saline (0.5% of Evans Blue); Veh: vehicle (20% DMSO in saline 0.5% of Evans Blue)

To investigate whether opioid or cannabinoid receptors were involved in PnPP-19-induced antinociception, the peptide was co-administered with non-specific and specific opioid antagonists and also with specific cannabinoid antagonists. As shown in Fig. 2, the intracerebroventricular administration of naloxone (2.5 and 5 μg) (Fig. 2a), clocinnamox (2 and 4 μg) (Fig. 2b), naltrindole (6 and 12 μg) (Fig. 2c) and AM251 (2 and 4 μg) (Fig. 2d) partially inhibited the antinociceptive response induced by 1 μg of PnPP-19. Taken together, these data suggest the participation of μ- and δ-opioid receptors and the CB_1_ cannabinoid receptor in the effect elicited by the peptide. The highest effective dose of the antagonists did not significantly modify the nociceptive threshold in control groups (Fig. 2a, b, c and d).

### Effect of nor-binaltorphimine and AM630 on PnPP19-induced antinociception

**Fig. 3:**
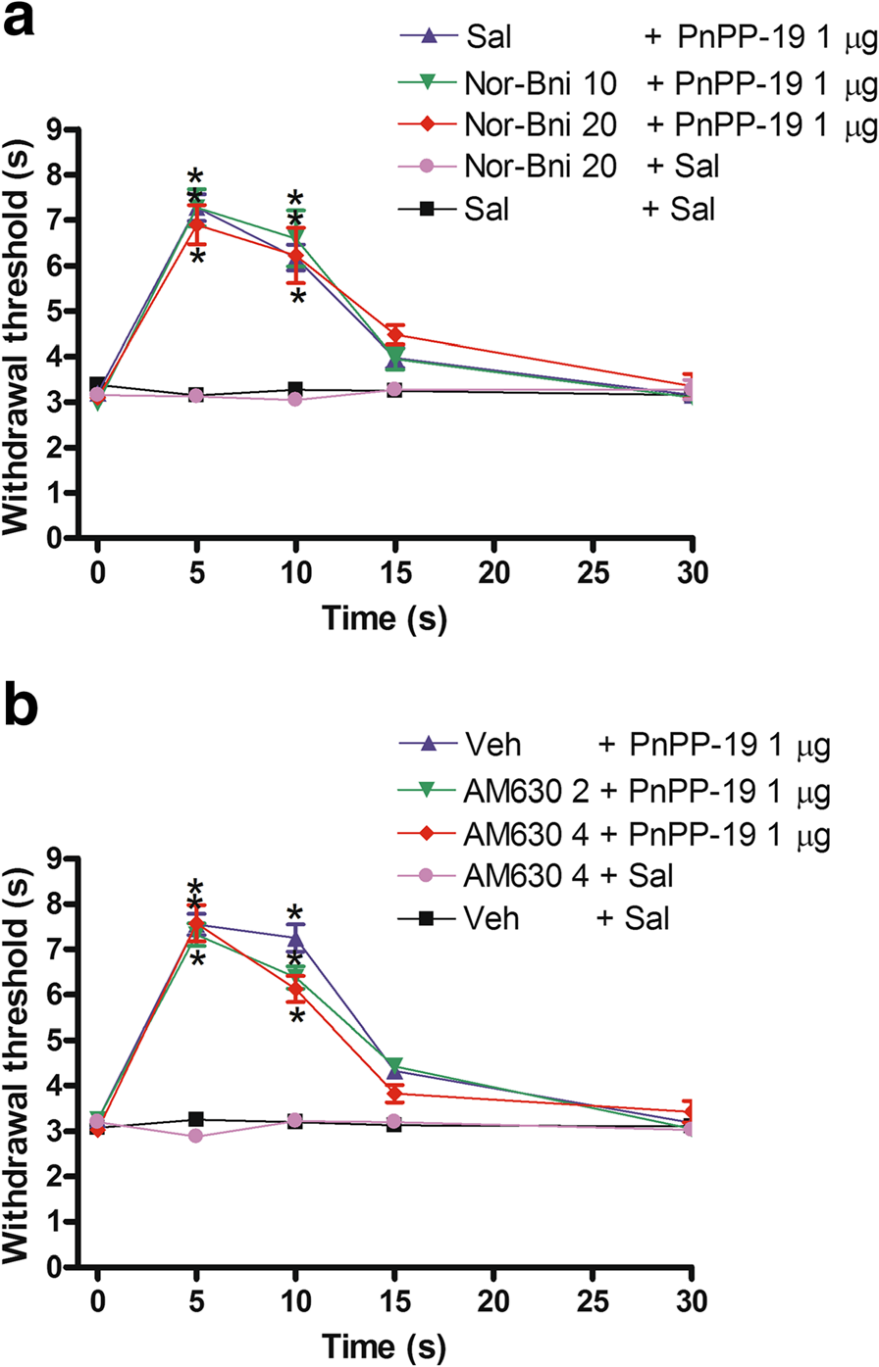
Intracerebroventricular administration of **a** nor-binaltorphimine or **b** AM630 on the central antinociception produced by PnPP-19. Nor-binaltorphimine (Nor-Bni; 10 and 20 μg) or AM630 (2 and 4 μg) was administered 1 min prior to PnPP-19 (1 μg) injection. These antagonists did not significantly modify the nociceptive threshold in the control group. Each line represents the mean ± SEM for four mice per group. *Significant difference compared to Sal + Sal or Veh + Sal injected groups (ANOVA + Bonferroni’s test). Sal: saline (0.5% of Evans Blue); Veh: vehicle (20% DMSO in saline 0.5% of Evans Blue)

The intracerebroventricular administration of nor-binaltorphimine (10 and 20 μg) and AM630 (2 and 4 μg) did not block the central antinociception of PnPP-19 (1 μg; Fig. 3a and b), suggesting that the activation of either κ-opioid receptors or CB_2_ cannabinoid receptors does not contribute to the peptide’s effect on the central nociceptive pathway. These drugs did not significantly modify the nociceptive threshold in control groups.

### Increase of PnPP-19-induced antinociception by bestatin, MAFP and VDM11

Because PnPP-19 induces activation of both opioid and cannabinoid receptors, we used the aminopeptidase inhibitor bestatin, the anandamide amidase inhibitor MAFP and the anandamide uptake inhibitor VDM11 to verify the possible involvement of the endogenous opioid and cannabinoid systems on PnPP-19-induced antinociception.

**Fig. 4:**
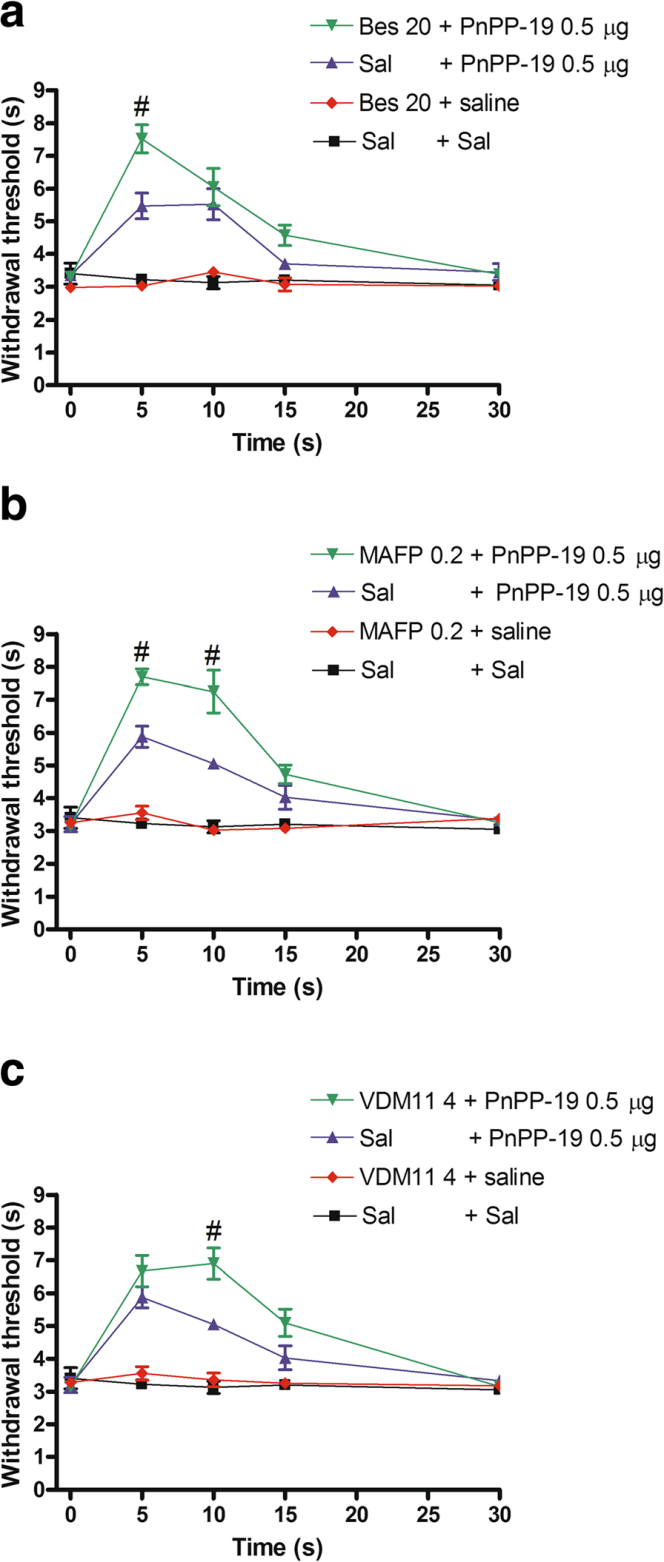
Potentiation of PnPP-19-induced antinociception by **a** bestatin, **b** MAFP or **c** VDM11. The bestatin (Bes; 20 μg), MAFP (0.2 μg) or VDM11 (4 μg) was administered 1 min prior to PnPP-19 (0.5 μg) injection. These drugs administered alone did not induce any effect. Each line represents the mean ± SEM for four mice per group. #Significant difference compared to the Sal + PnPP-19-injected group (ANOVA + Bonferroni’ test; *p* <0.05). Sal: saline (0.5% of Evans Blue)

In this experiment the PnPP-19 dose of 0.5 μg, instead of 1 μg, was employed to allow the observation of the potentiation effect that the selected inhibitors could induce. Therefore, at this time the ability of the aforementioned inhibitors to potentiate a lower dose of PnPP-19 (0.5 μg) was tested. Bestatin (20 μg, Fig. 4a), MAFP (0.2 μg, Fig. 4b) and VDM11 (4 μg, Fig. 4c) enhanced the antinociception induced by a low dose of PnPP-19 (0.5 μg). No significant modification of the nociceptive threshold was observed when bestatin, MAFP, VDM11 or vehicle were injected alone.

## Discussion

Spider venoms have been used as a potential source of new compounds with specific pharmacological properties. Some peptides extracted from the venom of the spider *Phoneutria nigriventer* have been suggested as potential sources of drugs for pain treatment. For instance, PnTx3-3 (ω ctenitoxin Pn2a) and Phα1β induce an antinociceptive effect in neuropathic pain models [4, 5]. More recently, we have shown that the synthetic peptide, PnPP-19, firstly characterized as a potentiator of erectile function, also produces antinociception in rats when peripherally injected [1, 17]. We also showed that this peripheral effect involves inhibition of neutral endopeptidase (NEP) (EC 3.4.24.11), and activation of CB_1_, μ- and δ-opioid receptors [17]. Therefore, the next issue to be investigated was whether PnPP-19 presents a possible central activity on nociception.

Our results demonstrate a dose-dependent central antinociceptive effect induced by PnPP-19 in the tail-flick test and reinforce the role of PnPP-19 as an analgesic drug candidate. We also investigated the possible participation of opioids and cannabinoids in the PnPP-19-induced central antinociception. In recent years, our group has shown the relationship between opioid and cannabinoid systems, as well as their involvement in the central and peripheral action mechanisms of different substances [9, 10, 15, 21, 22].

Interestingly, it was demonstrated that some animal toxins induce antinociception by activation of the opioid system. The analgesic effects of the neurotoxin from the king cobra’s venom (*Ophiophagus hannah*), the crude venom of the snake *Micrurus lemniscatus* and two scorpion toxins, AmmVIII (*Androctonus mauritanicus mauritanicus*) and LqqIT2 (*Leiurus quinquestriatus quinquestriatus*), were antagonized by administration of the opioid receptor antagonist naloxone [16, 23, 24]. Given the aforementioned information about the participation of opioids as at least part of the action mechanism of some toxins, and especially considering our previous results with PnPP-19 on peripheral nervous system, our experiments showed that naloxone partially inhibits the central antinociception induced by PnPP-19. As observed with a higher dose, a complete antagonism was not observed. This is the first report of opioid participation in the central antinociceptive mechanism of peptides derived from toxins purified from *Phoneutria nigriventer* venom.

Since naloxone interacts with μ-, κ- and δ-opioid receptors, highly selective antagonists were used to clarify which receptor subtype would be involved in the central antinociceptive effect of PnPP-19. Clocinnamox is an irreversible μ-opioid receptor antagonist with K_i_ values of 0.7, 1.9 and 5.7 nM for mouse μ-, δ- and κ-opioid receptors, respectively [25]. Naltrindole has 223- and 346-fold greater activity for δ- than for μ- and κ-opioid receptors, whereas nor-binaltorphimine shows 27- to 29-fold less potency, respectively, for μ and δ binding sites compared with κ receptors binding sites [26, 27].

Our results showed that clocinnamox and naltrindole, but not nor-binaltorphimine, partially antagonized PnPP-19-induced central antinociception, suggesting the participation of μ- and δ-opioid receptors in this effect, which is in accordance with previous findings on the peripheral nervous system [17]. In contrast, κ-opioid receptors appear to be involved in the antinociception induced by the crude venom of the snake *Micrurus lemniscatus* and the potent analgesic peptide isolated from the venom of the South American rattlesnake *Crotalus durissus terrificus*, crotalphine [24, 28]. The antinociception of crotalphine was blocked by pretreatment with selective antagonist of κ opioid receptors [28], an effect not observed in the present study when we tried to inhibit the antinociception of PnPP-19 by administration of a selective antagonist of the same opioid receptor.

In relation to opioid signaling, we applied the strategy of increasing the opioidergic system potency through opioid peptide catabolism inhibition. We observed that the administration of the aminopeptidase inhibitor bestatin significantly enhanced the central antinociception produced by a low dose of PnPP-19, providing evidence of the involvement of endogenous opioids in this effect. In vivo, enkephalins appear to be degraded by enzymes such as neutral endopeptidase and aminopeptidase [29]. Other opioid peptides, such as endorphin and dynorphin, appear to be resistant to neutral endopeptidase catabolism and, to a lesser extent, aminopeptidase [30].

Several studies have demonstrated reciprocal interactions between opioid and cannabinoid systems, suggesting a common underlying mechanism. For example, the cannabinoid Δ^9^-THC produces an increase in morphine antinociception by inducing the release of the endogenous opioid dynorphin [13]. On the other hand, the administration of the CB_1_ receptor antagonist AM251 inhibited morphine-induced antinociception [9, 15]. The synergy in the analgesic effects of these compounds is attributed to a crosstalk between these two signaling pathways mediated by simultaneous activation of opioid and cannabinoid receptors [31].

Recently, it was shown that peripheral interactions between cannabinoid and opioid systems contribute to the antinociceptive effect of the peptide crotalphine [32]. These authors demonstrated that crotalphine-induced antinociception stimulates local release of dynorphin A, which is dependent on CB_2_ receptor activation [32]. In contrast, we observed the participation of the CB_1_ receptor in PnPP-19-induced central antinociception, and as previously reported, μ and δ opioids receptors are also involved [17].

It has been suggested that CB_1_ and μ- and δ-opioid receptors form heterodimers [33]. These structures are necessary for the functioning of certain G-protein-coupled receptors, such as the GABA_B_ receptor [34]. A previous study demonstrated the important role for the heterodimer CB_1_-δ in neuropathic pain where cortical functions of δ opioid receptors were altered [35]. On the other hand, μ opioid receptors and CB_1_ receptors form a functional heterodimer and may transmit a signal through a common G protein mechanism [36].

As a consequence of this work, the identification of the endocannabinoid involved in pain modulation was assessed indirectly by administration of pharmacological agents that regulate uptake or degradation of anandamide. This endocannabinoid is an agonist of CB_1_ and CB_2_ receptors, but presents greater affinity for the former [37, 38]. The results demonstrated that the anandamide amidase inhibitor MAFP and anandamide uptake inhibitor VDM11 increase the central antinociception produced by a low dose of PnPP-19, suggesting the release of endocannabinoids and subsequent activation of CB_1_ receptors.

## Conclusions

In conclusion, our results show that PnPP-19 induces antinociception via the central nervous system and suggest that this effect is associated with the activation of μ−, δ − opioid and CB_1_ cannabinoid receptors. The release of endogenous opioids and endocannabinoids that might be acting on these receptors appears to be involved in the antinociceptive mechanism of the peptide. The results of this work contribute to elucidating the central antinociceptive effect of PnPP-19; however, more research is required to elucidate the interaction between opioid and cannabinoid systems in this effect.

In summary, our data together with the results obtained in the peripheral nervous system [17] show that PnPP-19 has a broad antinociceptive effect and thus constitutes a potential lead compound for the development of novel analgesic drugs.
